# 909. Clinical features and management of a human monkeypox outbreak: a retrospective observational study in Harlem New York

**DOI:** 10.1093/ofid/ofad500.954

**Published:** 2023-11-27

**Authors:** Raphael Shaw, Tjark Schliep, Vel Sivaplan, simona Bratu, Sharon Mannheimer, Donna Dowie, Mark Sayegh, Behnam Hajihossainlou, Erika Dickerson, Guerline Sinsmyr

**Affiliations:** Harlem Hospital, New York, New York; Harlem Hospital, New York, New York; Harlem Hospital, New York, New York; Harlem Hospital, New York, New York; Harlem Hospital Center/Columbia University, New York, New York; Harlem Hospital, New York, New York; Harlem Hospital, New York, New York; Harlem Hospital Center, NEW YORK, New York; Harem Hospital Center, NEW YORK, New York; Harem Hospital Center, NEW YORK, New York

## Abstract

**Background:**

In this paper, we report the descriptive, epidemiological, virologic and clinical features of the response to the investigational antiviral Tecovirimat, during the last Human Monkeypox outbreak in New City.

**Methods:**

This is a retrospective observational study, based on data obtained from a convenient sample of patients evaluated for monkeypox infection at Harlem Hospital Infectious Diseases Clinic, from June 30^th^ 2022 to September 1^st^ 2022. Outbreak analysis was completed using grid-like/mapping approach to evaluate the distribution of the lesions. Statistical analysis was conducted using binary logistic and Chi-square analysis.

Mapping of lesions

**Figure:**
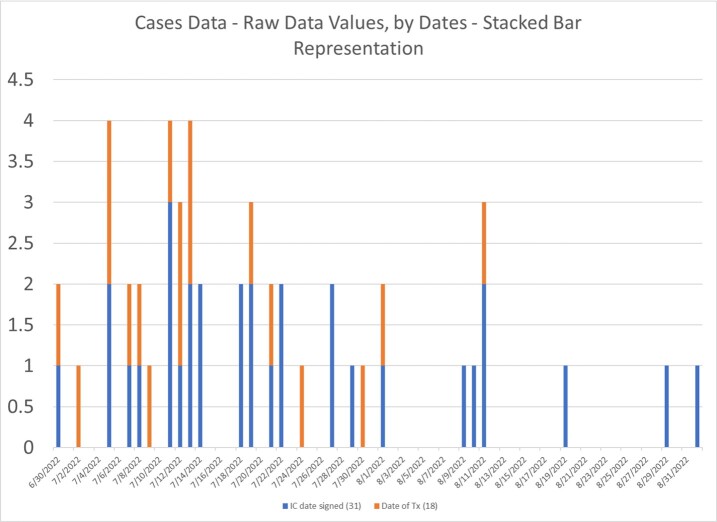

**Figure:**
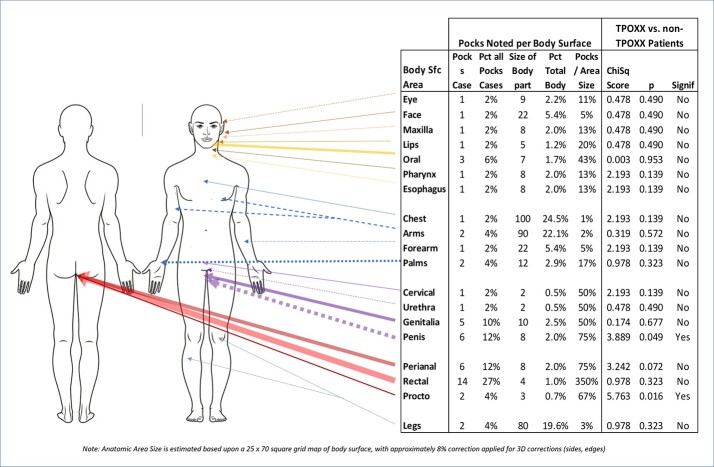

**Results:**

44 patients were included in the study 16 (36%) patients were immunocompromised with HIV with an absolute CD4 count ranging from 22 to 1048. Of the 44 patients, 30 (68%) were prescribed oral tecovirimat. Follow up showed that 13(43.3%) and 3 (3%) patients who received tecovirimat had complete resolution of symptoms at 7 days and 14 days respectively. 4 (9%) patients were lost to follow up and 2 (4.5%) were hospitalized for worsening symptoms. Overall medication was well tolerated with no reported events that were felt to be related to the medication.

Symptoms grid mapping showed a higher prevalence of lesions at the genital and rectal area, highly significant in the treatment group.

We were not able to establish a significant statistical correlation between HIV status/CD4 count and resolution of symptoms. However statistical analysis showed a significant positive correlation between treatment with Tecovirimat and resolution of symptoms at 14 days (P = 0.047).

**Conclusion:**

The results published in the abstract are mostly descriptive of the sample and the general management including the use of Tecovirimat.

Most patients who received tecovirimat had a complete resolution of symptoms n= (53%) at 14 days. It is not clear whether this difference was attributable to the efficacy of the treatment versus the natural course of disease. Indeed, several patients in the non-treatment group were lost to follow up.

Further studies are needed to define the effect of severe immunosuppression and the efficacy of available antiviral drugs on the course of disease.

**Disclosures:**

**All Authors**: No reported disclosures

